# Experimental and Numerical Investigation on the Aerosol Micro-Jet 3D Printing of Flexible Electronic Devices

**DOI:** 10.3390/ma16227099

**Published:** 2023-11-09

**Authors:** Yuanming Zhang, Tao Zhu, Junke Jiao, Shiyu Song, Zhenqian Wang, Ziwen Wang

**Affiliations:** 1 School of Mechanical and Vehicle Engineering, Linyi University, Linyi 276000, Chinawangzhenqian@lyu.edu.cn (Z.W.); 2 School of Automation and Electrical Engineering, Linyi University, Linyi, 276000, China; 210584001934@lyu.edu.cn (T.Z.);; 3 School of Mechanical Engineering, Yangzhou University, Yangzhou 225009, China

**Keywords:** aerosol micro-jet printing, additive manufacturing, flexible electronics, process research, simulation analysis, experimental verification

## Abstract

In this study, the optimal forming parameters for printing flexible circuits using aerosol jet printing technology are explored through numerical simulation and experiments. The printhead during the deposition process is numerically simulated. By employing the controlled variable method, the process parameters such as gas flow rate, working distance, nozzle diameter, and printing speed are selected to investigate their effects on the morphology of the printed lines. Accordingly, single-factor experiments are designed to validate the printing of flexible circuits on both planar and curved substrates. Laser micro-sintering is utilized to improve the conductivity of the printed lines and ultimately fabricate flexible strain sensors. Under the sheath gas flow rate of 400 sccm, carrier gas flow rate of 100 sccm, working distance of 3 mm, nozzle diameter of 500 μm, and printing speed of 10 mm/s, the optimal morphology of the printed lines is achieved with low linewidth characteristics. The variations in the focal ratio, working distance, nozzle diameter, and printing speed significantly affect the minimum feature line width and morphology of the printed lines.

## 1. Introduction

**Figure 1:**
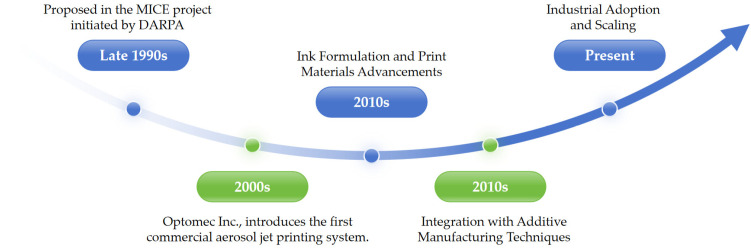
AMJP historical progression timeline.

Flexible electronics [1] is an emerging technology for building electronic devices on flexible or highly stretchable substrates. The key challenge in flexible electronics manufacturing is to efficiently print high-precision, micro-sized, and high-performance conductive circuits on flexible substrates. The traditional inkjet printing and screen printing technologies cannot meet the relevant requirements of high-precision and high-resolution printing. To this end, the emergence of aerosol micro-jet printing (AMJP) technology has effectively fulfilled the market demand for high-precision, high-resolution, low-cost, and multi-material flexible circuit printing. This technology utilizes inert gas to atomize liquid materials and print them as fine particles. It is characterized by high efficiency, viscosity, and non-contact features. The printing line accuracy can reach micrometer level, enabling high-resolution forming. It can also achieve conformal printing on complex surfaces. Consequently, it is widely used in microdevice fabrication, biomedical applications, and nanomaterial research [2,3,4,5,6,7], AMJP technology historical progression timeline as shown in Figure 1.

The flexible electronic devices can perform functions that conventional electronic devices cannot, including repeatable bending, rolling, stretching, and folding, which has increased their popularity in wide application markets. In particular, there has been a steep rise in the demand for flexible sensors in the medical and healthcare sector [8,9]. Although flexible sensors exhibit great potential in electronic skin and wearable electronic devices, their high-precision and high-quality fabrication still face some challenges [10]. The AMJP technology facilitates the direct printing of micrometer-level conductive circuits on flexible substrates, enabling high-resolution precision forming. However, the current process has some limitations, such as performance instability and complexity, which may lead to material deposition issues and poor device quality [11,12]. The phenomena like edge spreading caused by excessive spraying, occurrence of satellite droplets and deposition voids, and the discontinuous deposition at the edge of lines can deviate the additive manufacturing process from its optimal state. The existing studies on AMJP technology mainly focus on material preparation and processing. Theoretically, any material that can be suspended in aerosol after atomization is suitable for AMJP technology. This implies that advanced inks like two-dimensional (2D) materials and terahertz metamaterials can be printed using AMJP technology, thereby achieving high-performance microelectronic devices [13,14,15]. To address the performance issues of AMJP, Mahajan et al. [16] experimentally examined the effects of processing parameters, such as gas flow rate, nozzle diameter, and stage speed, on the width and thickness of printed lines as well as the performance of silver wires after sintering. Seifert et al. [17] presented a comprehensive comparison between inkjet printing and AMJP in terms of the deposited pattern elements, including single droplets, lines, and squares. Computational fluid dynamics (CFD) simulation often plays an important role in the process optimization and analysis. Salary et al. [18] established a computational platform to predict the aerosol flow regimes, ultimately achieving physics-driven in situ monitoring and control of the AMJP process. Chen et al. [19] developed a comprehensive 3D CFD model of the aerosol carrier gas flow. Using this model, the basic fluid mechanics principles controlling the overspray in the standard AMJP process were elucidated as a function of the droplet size distribution and the sheath gas flow rate.

Currently, there is limited research on AMJP technology, and the effect of process parameters on the formation and performance of flexible circuits is not comprehensive enough, with most of the studies focusing on planar substrates. To address these issues, in this study, the influence of different process parameters, such as gas flow rate, working distance, and nozzle diameter, on the AMJP process is investigated through numerical simulation and experimental verification. The impact of different parameters on the morphology of flexible circuits is examined using a physical model of aerosol jet printing. The main goal of this study is to obtain optimal AMJP parameters for printing conductive lines on both planar and curved substrates using flow field analysis and process parameter optimization, ultimately realizing the efficient fabrication of flexible strain sensors.

## 2. Materials and Methods

### 2.1. Experimental Materials

In this study, ZK-DryCure-Ag silver nanoparticle conductive ink (produced by Shandong Zhongke Intelligent Equipment Co., Ltd., Linyi, China) was used. The particle diameter was <50 nm, and the ink viscosity ranged between 0.004–0.01 Pa·s. The solid content of silver nanoparticles (mass fraction) was 10%. Dry high-pressure nitrogen gas was used as both the sheath gas and carrier gas. A polydimethylsiloxane (PDMS) substrate (Shandong Zhongke Intelligent Equipment Co., Ltd.) was used as the printing substrate. A substrate with a size of 15 mm × 15 mm was placed on a flat workbench, and a hemispherical substrate with a radius of 50 mm was placed on a five-axis workbench.

### 2.2. Experimental Equipment

A self-developed AMJP device was used in the experiment. The device consists of an aerosol generation module, a jet printing module, a motion control module, and a temperature control module. The aerosol generation module consists of a high-pressure nitrogen source, an ultrasonic atomization device, and a pneumatic atomization device. The jet printing module includes a three-axis motion platform, a five-axis slide table, a micro-jet printing head, and a laser micro-melting and solidification device, which can achieve printing on both planar and non-planar surfaces. The motion control module includes a motion control card and a computer. The temperature control module includes a nozzle heating rod, a heating atomizer, and a printing platform heating device for temperature control.

In the AMJP technology, the printing ink is atomized into small droplets through the atomization device in the aerosol generation module. Under the assistance of the carrier gas, which is an inert gas such as nitrogen, the atomized droplets are transported inside the printing nozzle. Under the influence of the sheath gas, which surrounds the interior of the nozzle, the multiphase aerosol flow is compressed and collimated. Finally, under the action of the three-axis motion platform or the five-axis slide table, the aerosol jet is deposited onto the planar or non-planar printing substrate to form high-precision and high-resolution conductive lines [20,21]. The choice of atomization device is usually determined by the ink properties. Ultrasonic atomization is used when the ink viscosity is 0.7–30 mPa·s and the particle diameter is <50 nm. Pneumatic atomization is chosen when the ink viscosity is 1–1000 mPa·s and the particle diameter is <0.5 μm [22]. In this experiment, the ink viscosity was 0.004–0.01 Pa·s and the particle diameter was <50 nm, so an ultrasonic atomization device was used. The microstructure of the conductive lines was analyzed using a CMY-290 trinocular metallographic microscope (Shangguang Instrument Co., Ltd., Suzhou, China).

### 2.3. Experimental Methods

The essence of the multiphase flow in AMJP is that the solid metal particles are suspended in a liquid solvent to form atomized droplets transported by the carrier gas. The aerosol jet flows inside the gas jetting head and is influenced by various factors. To simplify the theoretical model, the following assumptions are made: ① During the AMJP process, the aerosol and sheath gas are not compressed. ② In the aerosol jet, the volume fraction of nanoscale metal particles is less than 10% as compared to that of nitrogen gas, so a discrete-phase model is used for calculation. It is assumed that the velocity of the discrete-phase particles is the same as that of the continuous-phase particles, and the particles are non-rotating uniform spheres. ③ The proposed CFD model does not consider the interaction between the ink and substrate. ④ The Reynolds number within the gas jetting head is Re < 2300, and the sheath gas and aerosol jet are in a laminar flow state [23].

**Figure 2:**
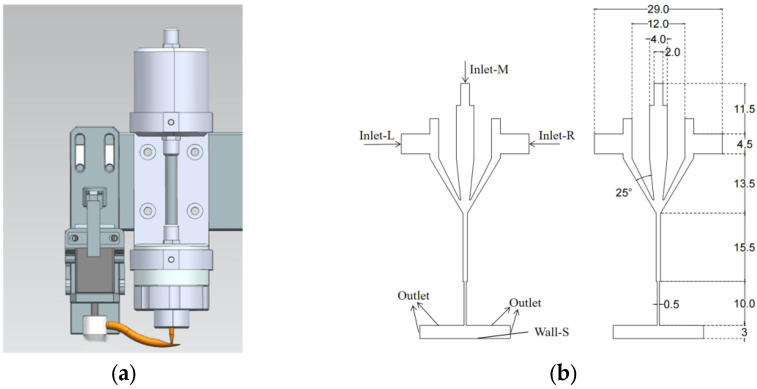
Micro-jet printing nozzle and computational fluid dynamics (CFD) model: (**a**) Micro-jet printing nozzle; (**b**) CFD model of micro-jet printing head (unit: mm).

Based on the micro-jet printing nozzle device shown in Figure 2a, a CFD model has been constructed using AutoCAD 2020 software, which is presented in Figure 2b. A 2D model of the fluid domain, consisting of the carrier gas inlet, sheath gas inlet, printing chamber, capillary nozzle, and independently fixed planar substrate, was established. In total, five types of boundaries were defined: carrier gas velocity inlet (Inlet-M), sheath gas velocity inlets (Inlet-L, Inlet-R), fixed wall (Wall), pressure outlet (Outlet), and curved substrate fixed wall (Wall-S).

In the CFD simulation, the density-based Navier–Stokes equations were used to solve the multiphase flow problem inside the micro-jet printing nozzle. The density field, velocity field, and temperature field were obtained using the continuity equation, momentum conservation equation, and energy conservation equation, respectively. The mass density and dynamic viscosity of nitrogen were calculated using the ideal gas state equation and the Sutherland formula, respectively. The Sutherland formula, which describes the shear viscosity of a gas, is shown as follows [24]:

(1) $$\mu =\mu_{0}{\left(\frac{T}{T_{0}}\right)}^{1.5}\frac{T_{0}+T_{S}}{T+T_{S}},$$

where μ represents the viscosity of the gas, $\mu_{0}$ represents the gas viscosity coefficient at $T_{0}$, $T_{0}$ represents the reference temperature (usually 273.15 K), and $T_{S}$ represents the Sutherland constant of the gas, which represents the strength of intermolecular collisions. The motion of aerosol droplets in shear flow is determined by Newton’s second law as follows:

(2) $$\sum {\vec{F}}_{drop}=V_{d}\rho_{d}\frac{d\vec{v}}{dt},$$

where ${\vec{F}}_{drop}$ represents the total external force acting on the aerosol droplets; $V_{d}$, $\rho_{d}$, and $\vec{v}$ represent the volume, density, and velocity vector of the aerosol droplets, respectively. The sum of all the external forces acting on the aerosol droplets in shear flow can be expressed as follows:

(3) $$\sum {\vec{F}}_{drop}={\vec{F}}_{Ba}+{\vec{F}}_{Bu}+{\vec{F}}_{DF}+{\vec{F}}_{g}+{\vec{F}}_{ML}+{\vec{F}}_{PG}+{\vec{F}}_{SL}+{\vec{F}}_{VM},$$

where ${\vec{F}}_{Ba}$ represents the Basset force, ${\vec{F}}_{Bu}$ represents the buoyancy force, ${\vec{F}}_{DF}$ represents the drag force, ${\vec{F}}_{g}$ represents the gravitational force,${\vec{F}}_{ML}$ represents the Magnus lift force, ${\vec{F}}_{PG}$ represents the pressure gradient force, ${\vec{F}}_{SL}$ represents the Saffman lift force, and ${\vec{F}}_{VM}$ represents the virtual mass force. Based on the aforementioned assumptions, only the drag force and Saffman lift force of the aerosol droplets are considered in this discrete phase model. The drag force experienced by the particles is calculated using the Stokes drag model, and the relevant formula is shown below:

(4) $${\vec{F}}_{DF}=6\pi \eta \left|\vec{u}-\vec{v}\right|R,$$

where η represents the dynamic viscosity of the fluid, R represents the particle radius, and $\left|\vec{u}-\vec{v}\right|$ represents the relative velocity of particles in the fluid. For particles moving in laminar flow, the Stokes equation can be rewritten as follows:

(5) $${\vec{F}}_{DF}=3\pi \mu_{d}df\left|\vec{u}-\vec{v}\right|,$$

where $\mu_{d}$ represents the dynamic viscosity of the fluid, *d* represents the particle diameter, and f represents the drag coefficient. The Saffman lift force acting on the aerosol droplets, which represents the lift experienced by the atomized droplets moving in the carrier gas, is expressed as follows:

(6) $${\vec{F}}_{SL}=1.61d^{2}\left|\vec{u}-\vec{v}\right|\sqrt{\mu_{d}\rho \frac{d\vec{u}}{dy}},$$

where ρ represents the density of the aerosol droplet, and $\vec{u}$ represents the velocity vector of the continuous phase. During the micro-jet printing process, the Saffman lift force acts in the direction perpendicular to the particle’s motion, redirecting the atomized droplets towards the centerline of the nozzle where the fluid velocity is higher.

Using the self-developed AMJP device, conductive circuits were printed on a PDMS substrate at ambient temperature to study the effects of different carrier gas flow rates, sheath gas flow rates, focusing ratios, working distances, and nozzle diameters on the AMJP performance. By keeping the sheath gas flow rate at 100 sccm, working distance at 3 mm, nozzle diameter at 500 μm, and printing speed at 10 mm/s, the carrier gas flow rate was varied to investigate its impact on the conformal printing on curved surfaces. Similarly, the effects of sheath gas flow rate, working distance, and nozzle diameter on AMJP were studied using a controlled variable method. The experimental factors for AMJP are detailed in Table 1. To facilitate the analysis of the gas flow rate’s influence, the focusing ratio, denoted as X, is defined as follows:

(7) $$X=\frac{Q_{S}}{Q_{C}},$$

where $Q_{S}$ represents the sheath gas flow rate, and $Q_{C}$ represents the carrier gas flow rate.

### 2.4. Result Characterization Processing

This study focuses on the impact of process parameters on the AMJP performance. The internal aerosol flow in the micro-jet printing nozzle is the main object of study, and a combined approach of simulation analysis and experimental verification is used. During the simulation, the key positions of gas flow are at the convergence of sheath gas and carrier gas and at the nozzle outlet. Therefore, these two positions are mainly considered in the simulation analysis. The variation in the working distance mainly manifests in the variation of particle trajectories between the nozzle outlet and the curved substrate. Therefore, the particle trajectories between the outlet and the substrate are mainly considered in the relevant simulation analysis. The variation in nozzle diameter affects the flow of the aerosol beam entering the capillary nozzle and the flow ejected from the nozzle outlet. Therefore, the entrance of the capillary nozzle and the outlet of the AMJP nozzle are mainly considered in the simulation analysis of nozzle diameter.

**Figure 3:**
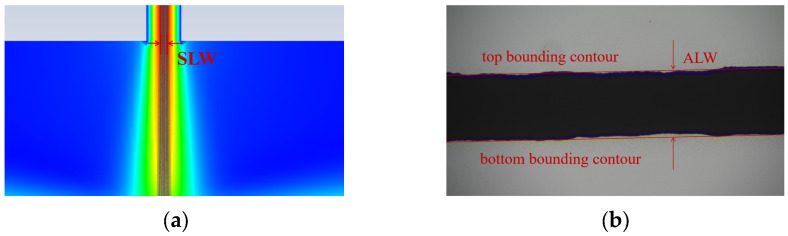
Judgment criteria for the simulated minimum feature line width (SLW) and the actual minimum feature line width (ALW): (**a**) SLW; (**b**) ALW.

The diameter of the aerosol beam at the nozzle outlet obtained from the simulation is selected as the simulated minimum feature line width (SLW), as shown in Figure 3a. The evaluation criterion proposed by Binder et al. [25] is used to determine the minimum feature line width. The particle region exceeding 50% of the maximum packing density of particles is considered as the determination criterion for the actual minimum feature line width (ALW), and the influence of edge satellite droplets on the measurement of the minimum feature line width is ignored, as shown in Figure 3b.

## 3. Results

### 3.1. Physical Model Analysis

**Figure 4:**
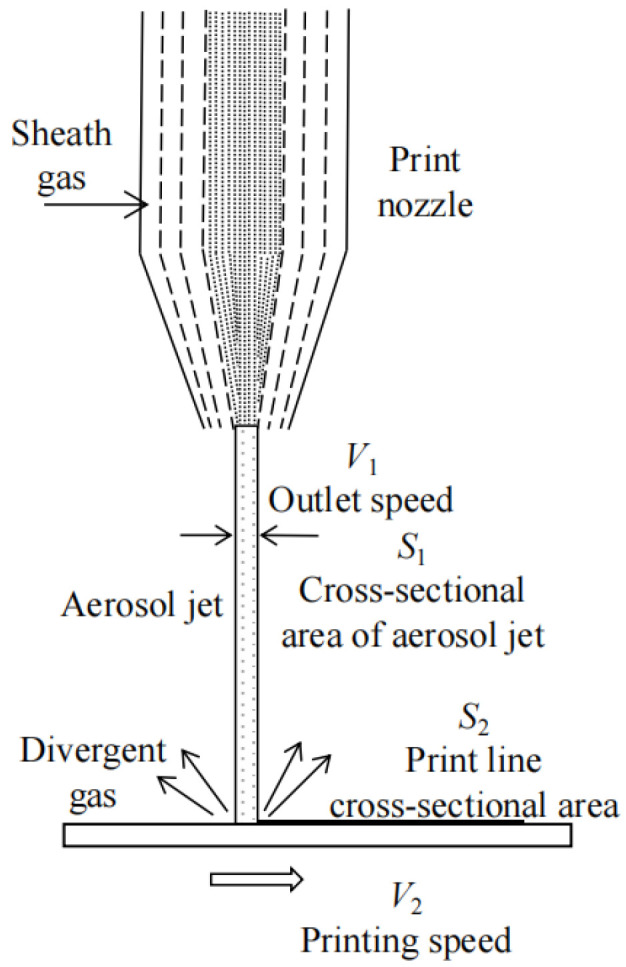
Physical principle of AMJP.

Figure 4 illustrates the physical characteristics of the aerosol jet stream from the nozzle to the printing substrate in AMJP. The sheath gas surrounds the aerosol beam, preventing nozzle clogging and facilitating collimation and compression of the aerosol beam. At the nozzle outlet of the printing head, when the sheath gas and aerosol beam pass through a narrow nozzle opening, the atomized droplets transported by the carrier gas are further focused. The multiphase flow of aerosol is accelerated and directed toward the nozzle outlet, resulting in the ejection of the aerosol jet stream at the nozzle mouth.

In the absence of material loss and solvent evaporation from the printed lines, considering the mass flow conservation between the nozzle outlet and the impact of the aerosol jet stream on the substrate, the continuity equation is applicable [26], i.e.,

(8) $$\rho_{1}S_{1}V_{1}=\rho_{2}S_{2}V_{2}+m_{g},$$

where $S_{1}$ and $V_{1}$ represent the cross-sectional area and velocity at the nozzle outlet of the aerosol jet stream, while $S_{2}$ and $V_{2}$ represent the cross-sectional area and movement speed of the printed lines on the substrate. $\rho_{1}$ and $\rho_{2}$ represent the densities of the aerosol jet stream and the material of the printed lines, respectively. $m_{g}$ represents the mass flow rate of the divergent gas deposited onto the substrate. According to the relationship between the flow rate and velocity [27], it can be obtained that:

(9) $$Q_{n}=V_{1}S_{1}=V_{1}\frac{\pi d^{2}}{4},$$

where $Q_{n}$ represents the gas flow rate at the nozzle outlet, and d represents the nozzle diameter. Combining Equations (8) and (9), as follows:

(10) $$S_{2}=\frac{\rho_{1}S_{1}V_{1}-m_{g}}{\rho_{2}V_{2}}=\left(\frac{4\rho_{1}}{\pi \rho_{2}}\right)\left(\frac{S_{1}Q_{n}}{V_{2}d^{2}}\right)-\frac{m_{g}}{\rho_{2}V_{2}},$$

where w represents the minimum feature line width of the printed lines, and t represents the thickness of the printed lines. 

Based on the experimental conditions, when the carrier gas flow rate $Q_{C}$ is kept constant, an increase in the focusing ratio X can cause an increase in the sheath gas flow rate $Q_{S}$. This enhances the compression effect of the sheath gas on the aerosol beam, leading to a decrease in the cross-sectional area $S_{1}$ at the nozzle outlet. Assuming that $\rho_{1}$ remains constant, the decrease in $S_{1}$ can also cause an increase in the velocity $V_{1}$ at the nozzle outlet, while keeping their product $S_{1}V_{1}$ constant. Assuming that $\rho_{2}$ does not vary significantly under different values of X, Equation (10) implies that the cross-sectional area $S_{2}$ of the printed lines remains constant. Further, the cross-sectional area $S_{2}$ can be approximated as the product of line width w and line thickness t. The experimental results suggest that an increase in X leads to a decrease in the minimum feature line width w, indicating an increase in the thickness t of the lines. The effects of working distance, nozzle diameter, and printing speed can be analyzed in a similar manner.

### 3.2. Gas Flow Rate

**Figure 5:**
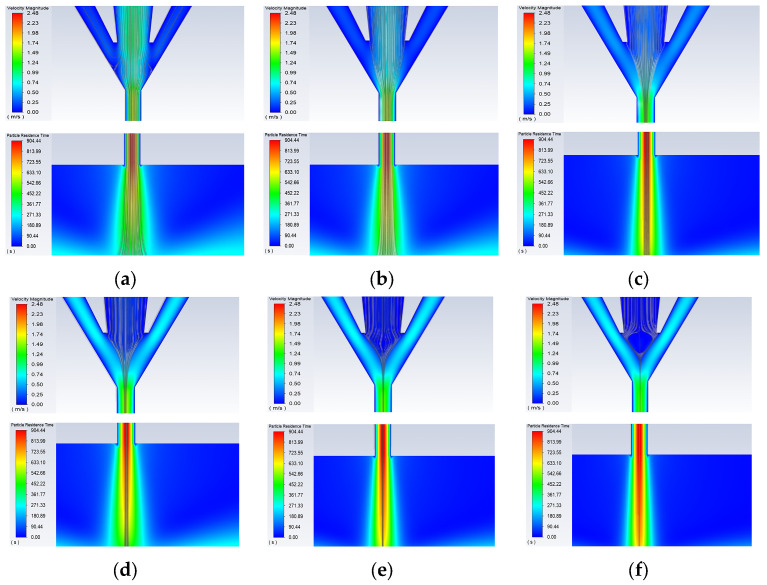
Simulation images of the internal nozzle at different sheath gas flow rates: (**a**) *Qs* = 50 sccm; (**b**) *Qs* = 100 sccm; (**c**) *Qs* = 200 sccm; (**d**) *Qs* = 300 sccm; (**e**) *Qs* = 400 sccm; (**f**) *Qs* = 500 sccm.

**Figure 6:**
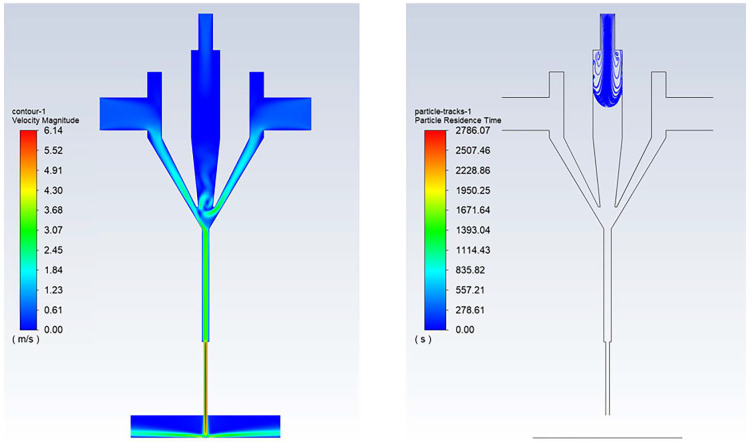
Simulation image at a sheath gas flow rate of 600 sccm.

First, keeping the carrier gas flow rate at 100 sccm, working distance at 3 mm, nozzle diameter at 500 μm, and printing speed at 10 mm/s, the sheath gas flow rate $Q_{S}$ was varied to 50, 100, 200, 300, 400, 500, and 600 sccm, with the focusing ratio *X* set to 0.5, 1, 2, 3, 4, 5, and 6 for simulation analysis. The simulation results are shown in Figure 5 and Figure 6. It can be seen that as the sheath gas flow rate increases, the compression effect of the sheath gas inside the aerosol printing chamber gradually strengthens, causing a reduction in the width of the aerosol jet stream. When the sheath gas flow rate is low (50 sccm), the compression effect of the sheath gas at the airflow convergence in the aerosol printing chamber is not significant. As a result, a small portion of the atomized droplets enters the sheath gas inlet along with the carrier gas, and the sheath gas does not provide noticeable focusing and collimation effects. This leads to a wide diameter of the aerosol jet stream at the nozzle outlet (Figure 5a). As the sheath gas flow rate gradually increases, the focusing effect of the sheath gas becomes more pronounced, leading to a decrease in the diameter of the aerosol jet stream. When the sheath gas flow rate becomes too large (nearly 600 sccm) with a focusing ratio of X = 6, the carrier gas becomes unable to transport the atomized droplets into the printing chamber. Consequently, the aerosol jet stream cannot be ejected, as shown in Figure 6.

**Figure 7:**
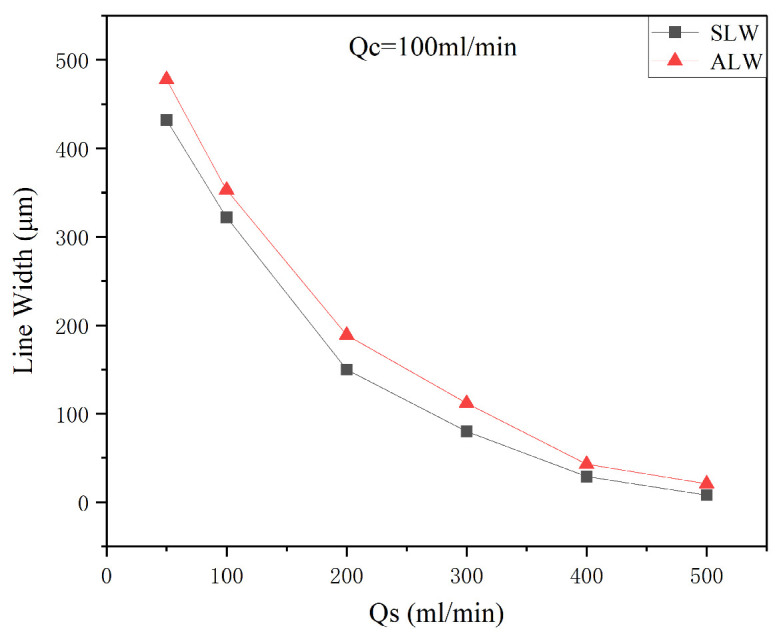
Variation in the minimum feature line width as a function of gas flow rate.

Next, under a carrier gas flow rate of 100 sccm, a working distance of 3 mm, a nozzle diameter of 500 μm, and a printing speed of 10 mm/s, the effect of different sheath gas flow rates ($Q_{S}$ = 50, 100, 200, 300, 400, and 500 sccm) on the minimum feature line width was investigated. A comparison between the experimental results and simulation data is shown in Figure 7. Under a constant carrier gas flow rate of 100 sccm, an increase in the sheath gas flow rate results in a higher gas flow focusing ratio. Both the SLW and ALW decrease with the increase in focusing ratio. It should be noted that the SLW values are lower than the ALW values. This can be attributed to the unavoidable physical scattering and diffusion phenomena that occur during the movement of the aerosol beam from the nozzle to the curved substrate and during its deposition on the curved substrate. These phenomena gradually increase the diameter of the aerosol beam in the experiment.

**Figure 8:**
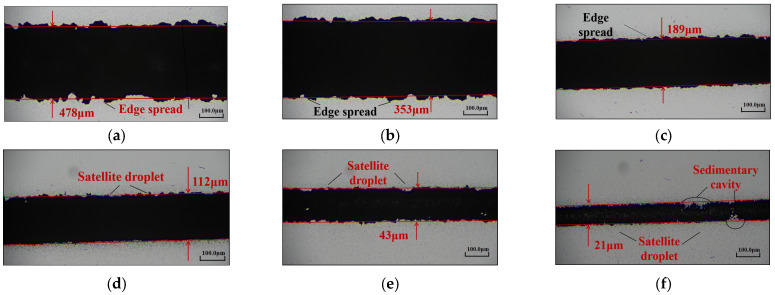
Line morphology at different focusing ratios with a fixed carrier gas flow rate: (**a**) *X* = 0.5; (**b**) *X* = 1; (**c**) *X* = 2; (**d**) *X* = 3; (**e**) *X* = 4; (**f**) *X* = 5.

The morphology of the line features was analyzed using an optical microscope, and the results are shown in Figure 8. It is clear that when the focusing ratio X = 0.5, the minimum feature line width of the printed lines is relatively large, and the lines exhibit overspray phenomenon. The multiphase flow of aerosol inside the nozzle includes carrier gas flow, atomized solvent droplets, and silver nanoparticles encapsulated within the solvent droplets. When X is small and the sheath gas flow rate $Q_{S}$ is low, the compression effect of the sheath gas is not significant, resulting in a larger cross-sectional area S1 of the aerosol multiphase flow and the deposition of more aerosol droplets on the substrate. This leads to the formation of densely packed silver particles in the middle of the printed lines and the diffusion of solvent droplets toward the edges due to their flow characteristics, causing overspray during printing. As the sheath gas flow rate gradually increases, the compression and collimation effects of the sheath gas become more pronounced. The aerosol jet stream deposited onto the curved substrate converges toward the center of the printed lines, causing a significant reduction in the minimum feature line width and suppression of the edge spreading phenomenon. When X = 4, the line width decreases significantly, accompanied by only slight edge spreading and the appearance of satellite droplets (Figure 8f). The satellite droplets appear when both X and the sheath gas flow rate $Q_{S}$ are large. The cross-sectional area of the aerosol multiphase flow at the nozzle outlet is smaller, and since the carrier gas flow rate remains constant, the outlet velocity increases, causing particle splashing and the formation of satellite droplets on the substrate. When X = 5, the satellite droplet phenomenon becomes severe, and due to the large value of X and $Q_{S}$, the droplets deposited on the substrate do not contain silver nanoparticles. As the solvent evaporates, voids are formed, resulting in the deposition void phenomenon (Figure 8f).

**Figure 9:**
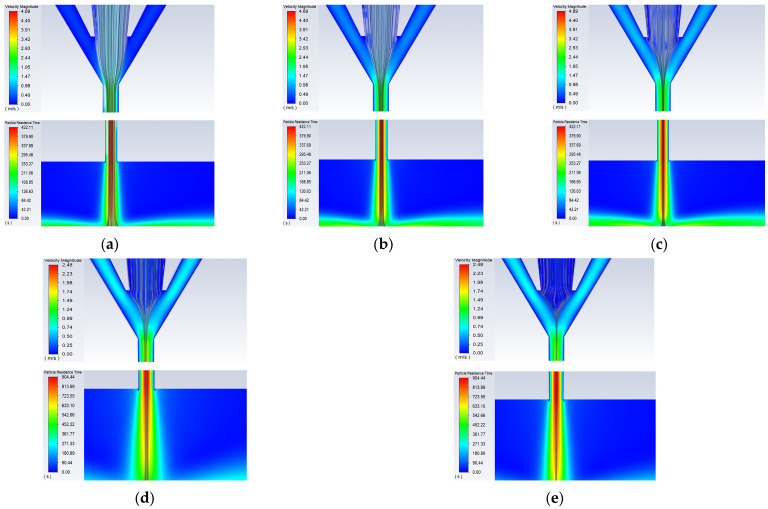
Simulation results at different focusing ratios when the pressure is constant: (**a**) *X* = 1; (**b**) *X* = 1.5; (**c**) *X* = 2; (**d**) *X* = 2.5; (**e**) *X* = 3.

The above analysis was based on varying the focusing ratio while keeping the carrier gas flow rate constant and changing the sheath gas flow rate. However, as the focusing ratio changes, the total flow rate of carrier and sheath gases also changes, resulting in different pressures at the nozzle outlet under different focusing ratios. Therefore, a method is proposed to maintain a constant total gas flow rate while simultaneously changing the carrier gas flow rate and sheath gas flow rate to investigate the effect of the focusing ratio on the printed line morphology. Under a constant total gas flow rate of 500 sccm, simulations were conducted at focusing ratios of 1, 1.5, 2, 2.5, and 3. The simulation results are shown in Figure 9. It can be seen that as the focusing ratio *X* increases, the sheath gas flow rate gradually increases, while the carrier gas flow rate decreases. Consequently, the SLW of the aerosol jet shows a decreasing trend, which is similar to the trend observed when the total gas flow rate changes.

In conclusion, as X increases, w shows a decreasing trend, indicating that an increase in the gas flow focusing ratio is conducive to improving the accuracy of printed lines.

### 3.3. Nozzle Diameter

**Figure 10:**
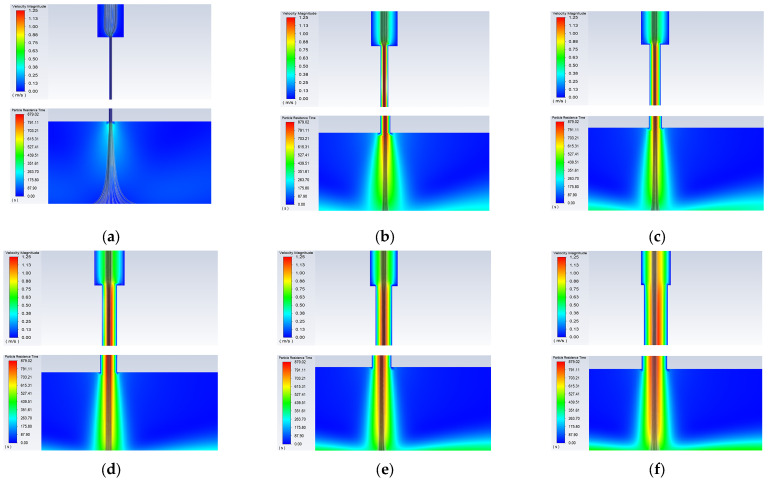
Simulated images of the internal nozzle at different nozzle diameters: (**a**) *d* = 100 μm; (**b**) *d* = 300 μm; (**c**) *d* = 400 μm; (**d**) *d* = 500 μm; (**e**) *d* = 600 μm; (**f**) *d* = 800 μm.

Simulations were conducted under a fixed carrier gas flow rate of 100 sccm, sheath gas flow rate of 400 sccm, working distance of 3 mm, printing speed of 10 mm/s, and the nozzle diameters were varied to 100, 300, 400, 500, 600, and 800 μm. The simulation results of particle trajectories and velocities are shown in Figure 10. It can be seen that when the nozzle diameter is small (100 μm), very few aerosol droplets enter the capillary nozzle, resulting in a narrow diameter of the aerosol jet emitted from the nozzle. As the nozzle diameter increases, more aerosol droplets enter the capillary nozzle, and the diameter of the aerosol jet emitted from the nozzle also increases.

**Figure 11:**
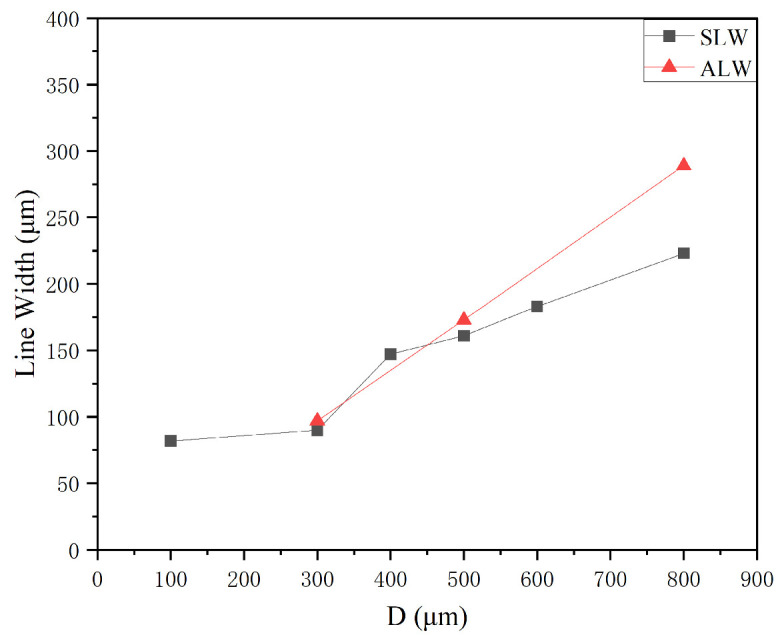
Variation in the minimum feature line width as a function of nozzle diameter.

According to the simulation results, experiments were conducted by varying the nozzle diameter to 300, 500, and 800 μm under a sheath gas flow rate of 400 sccm, carrier gas flow rate of 100 sccm, working distance of 3 mm, and printing speed of 10 mm/s. The simulation and experimental results are compared in Figure 11. It is clear that as the nozzle diameter increases, the minimum feature line width gradually increases. The variation trend of SLW is consistent with that of ALW, and SLW is always lower than ALW. 

**Figure 12:**
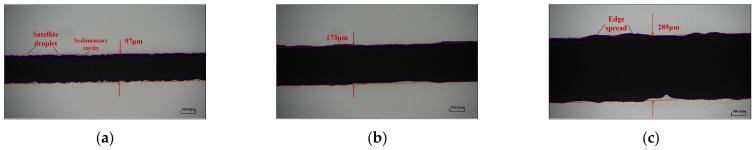
Line morphology at different nozzle diameters: (**a**) *d* = 300 μm; (**b**) *d* = 500 μm; (**c**) *d* = 800 μm.

The optical microscopy images depicting the line morphology are shown in Figure 12. It can be seen that when the nozzle diameter is 300 μm, the small nozzle exit diameter leads to a high pressure at the exit, resulting in a higher aerosol jet speed and fewer atomized droplets transported by the carrier gas. Consequently, when a curved substrate is used for deposition, splashing of droplets occurs, leading to the appearance of satellite droplets and deposition voids. As the nozzle diameter increases, the pressure at the nozzle exit gradually decreases, the aerosol jet speed decreases, and more atomized droplets are ejected. Further, the cross-sectional area of the aerosol jet increases, leading to a larger value of the minimum feature line width deposited on the curved substrate. When the diameter increases to 800 μm, overspray with edge spreading phenomenon occurs.

### 3.4. Working Distance

**Figure 13:**
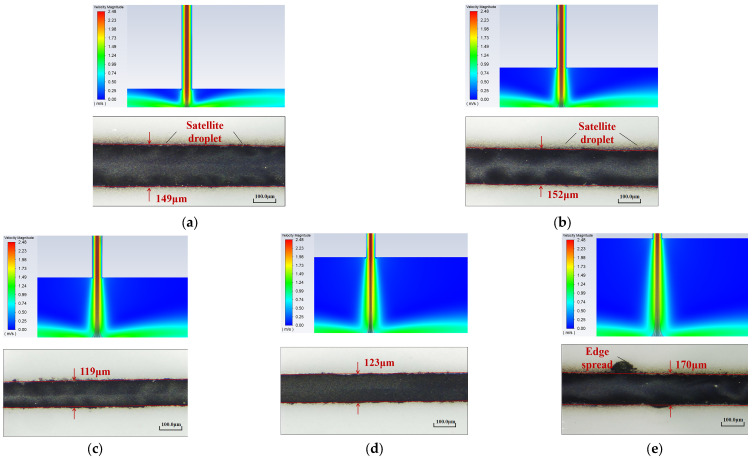
Simulation images and line morphologies of the internal nozzle at different working distances: (**a**) *L* = 1 mm; (**b**) *L* = 2 mm; (**c**) *L* = 3 mm; (**d**) *L* = 4 mm; (**e**) *L* = 5 mm.

Simulations and experiments were conducted by varying the working distance to 1, 2, 3, 4, and 5 mm while keeping the carrier gas flow rate at 100 sccm, sheath gas flow rate at 400 sccm, nozzle diameter at 500 μm, and printing speed at 10 mm/s. The simulation and experimental results for the line morphologies are shown in Figure 13. It can be observed that when the working distance is between 1–2 mm, i.e., the distance between the nozzle and the curved substrate is too small, the sheath gas cannot exert its intended collimation and compression effects. Additionally, the initial velocity of the ejected droplets is too high, resulting in a high velocity of the aerosol jet deposited on the substrate, leading to droplet splashing and the formation of satellite droplets. As the working distance gradually increases, the sheath gas has sufficient space to exert its effects, thereby suppressing the occurrence of satellite droplets. However, when the working distance increases to 5 mm, the distance between the nozzle and the curved substrate becomes too large, causing the sheath gas to weaken. This leads to inevitable physical scattering of the aerosol jet and the occurrence of overspray with edge spreading on the printing substrate.

**Figure 14:**
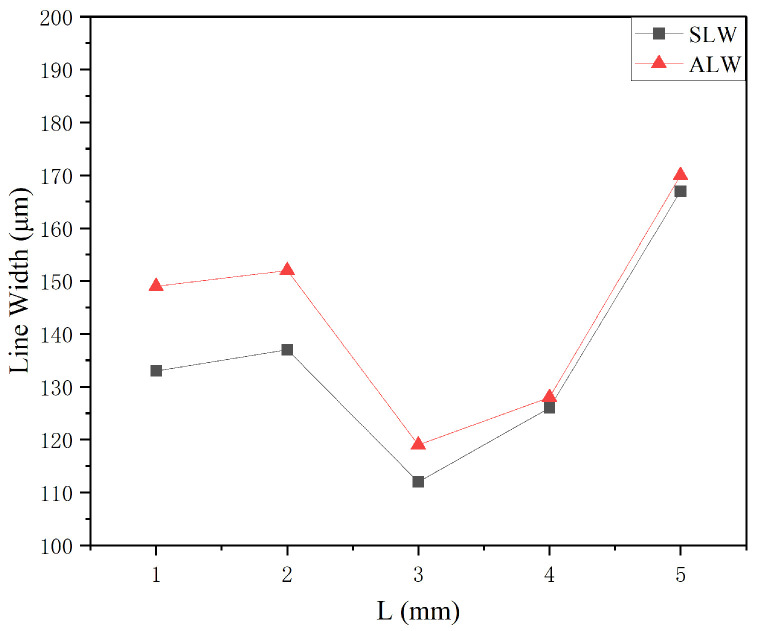
Variation in the minimum feature line width as a function of working distance.

The impact of the working distance on the printing process is mainly reflected in the contact position between the aerosol jet and the substrate. Therefore, the width of the particle trajectory deposited on the substrate in the simulation results is selected as the SLW. The SLW is compared with the ALW in Figure 14. It is clear that the values of the minimum feature line width generally increase with the increase in the working distance. The SLW and ALW show a similar trend, but the SLW values are lower than the ALW values. This is because the predicted value is the width of the particle trajectory when it contacts the printing substrate, but in the actual experiment, the particles deposited on the curved substrate inevitably undergo flow diffusion and ejection with the transporting gas. When the working distance is less than 2 mm, the sheath gas does not have enough space to exert the constraining effect, resulting in insufficient compression of the aerosol jet width. Additionally, the high initial velocity at the nozzle exit causes splashing of the ejected droplets on the substrate, leading to slightly higher line width values. When the working distance increases to 2–3 mm, the constraining effect of the sheath gas gradually becomes evident, and the width of the printed lines shows a temporary decrease. When the working distance becomes very high (exceeds 3 mm), the constraining effect of the sheath gas weakens, and the aerosol droplets ejected onto the substrate spread extensively, resulting in wider printed lines and a significant increase in the minimum feature line width on the substrate.

Overall, as the working distance increases, the minimum feature line width of the printed lines tends to increase, suppressing the occurrence of satellite droplets but gradually leading to significant overspray with edge spreading. At L = 3 mm, the printed line morphology is improved, and the minimum feature line width is the lowest.

### 3.5. Printing Speed

**Figure 15:**
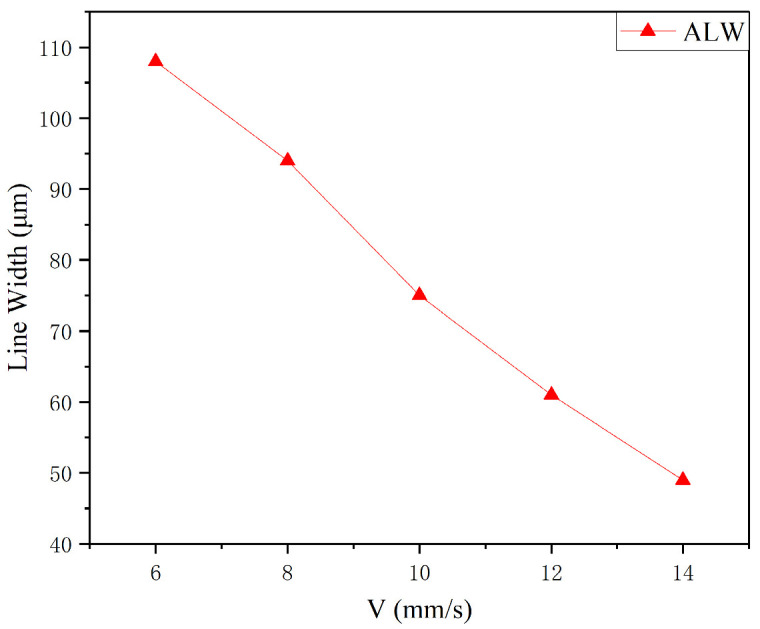
Variation in the minimum feature line width as a function of printing speed.

Under a focal ratio of 4, a working distance of 3 mm, and a nozzle diameter of 500 μm, the effect of different printing speeds (6, 8, 10, 12, and 14 mm/s) on the line morphology was investigated. Since the numerical simulations were conducted using a 2D model, the simulation analysis of printing speed was not performed. Instead, preliminary experiments were conducted to analyze the appropriate range of printing speeds, which were then selected for the experimental analysis. In the future, a 3D model will be utilized to simulate the printhead and investigate the effect of printing speed. The variation in the minimum feature line width as a function of the printing speed is shown in Figure 15. It is clear that as the printing speed increases, the minimum feature line width of the printed lines tends to decrease.

**Figure 16:**
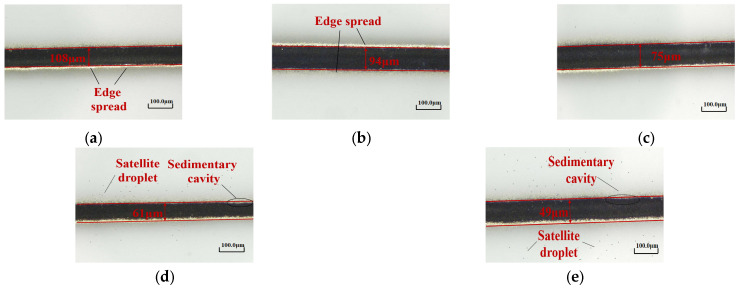
Line morphology at different printing speeds: (**a**) V = 6 mm/s; (**b**) V = 8 mm/s; (**c**) V = 10 mm/s; (**d**) V = 12 mm/s; (**e**) V = 14 mm/s.

Figure 16 shows the characteristic line morphologies obtained at different printing speeds under an optical microscope. When V = 6 mm/s (Figure 16a), due to the slow printing, a higher amount of aerosol jet is deposited on the substrate, resulting in the formation of a dense silver line in the middle of the line. Due to its flow characteristics, the ink solvent tends to deposit more at the edges, leading to overspray phenomena on the edges. When V increases to 8 mm/s (Figure 16b), the minimum feature line width of the printed lines gradually decreases, and the overspray phenomenon at the edges is slightly alleviated. When V = 10 mm/s (Figure 16c), the overspray phenomenon is significantly improved. When V = 12 mm/s (Figure 16d), the minimum feature line width continues to decrease, and satellite droplets start to appear, accompanied by slight deposition voids. With further increase in the printing speed to V = 14 mm/s (Figure 16e), the minimum feature line width decreases to approximately 49 μm, and satellite droplets and deposition voids become apparent. This is because when the other influencing factors remain constant, the high printing speed causes a reduction in the deposition of silver nanoparticles on the substrate, leading to splashing phenomena.

Based on the above experimental analysis, it can be concluded that as the printing speed increases, the minimum feature line width of the printed lines gradually decreases, suppressing overspray phenomena such as edge spreading. However, it also leads to the gradual occurrence of satellite droplets and deposition voids. At V = 10 mm/s, the minimum feature line width is low, and the line morphology is optimal.

### 3.6. Printing of Flexible Circuit

**Figure 17:**
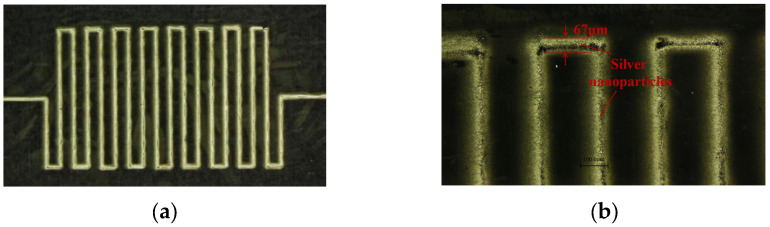
Flexible circuit printed on flat substrate: (**a**) electronic circuit; (**b**) microstructure.

Based on the above experimental analysis, to eliminate the deposition voids produced during single-layer printing and increase the density of silver nanoparticles in the middle of the printed lines, the following parameters are selected: sheath gas flow rate = 400 sccm, carrier gas flow rate = 100 sccm, *L* = 3 mm, *d* = 500 μm, and V = 10 mm/s, and the electronic circuit is printed by stacking 10 layers, as shown in Figure 17a. The optical microscopy images of the line morphology reveal that the final printed line width is 67 μm. It is clear that the printed lines exhibit a higher density level of silver nanoparticles in the center and a lower density level on the sides of the substrate, as shown in Figure 17b. This is attributed to the influence of the Saffman lift, which causes the nanoparticles in motion at the nozzle to gather in the center. Consequently, there is an excess of silver nanoparticles in the center as compared to the edges.

**Figure 18:**
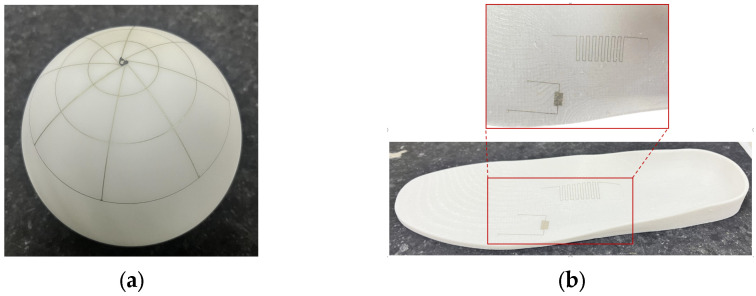
Flexible circuit printed on curved substrates: (**a**) the conformal printing of a flexible circuit on a curved substrate; (**b**) printing conductive lines on 3D-printed insoles.

Based on the optimal process parameters and using silver nanoparticle ink as the printing material, the self-developed aerosol jet printing device was successfully utilized for the conformal printing of a flexible circuit on a curved substrate. The result is shown in Figure 18a. Furthermore, conductive circuits were printed on a 3D-printed insole, achieving conformal printing of electronic circuits on a complex curved surface, as shown in Figure 18b. The accuracy and quality of the printed lines are much better than those obtained using the traditional inkjet printing technology. Therefore, the AMJP technology is highly suitable for the fabrication of flexible electronic devices and can also be used on irregular and complex surfaces.

### 3.7. Fabrication of Flexible Strain Sensors

**Figure 19:**
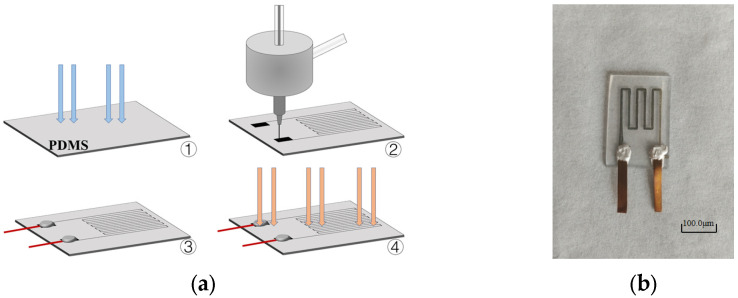
Fabrication of flexible strain sensors: (**a**) Printing process of flexible strain sensor; (**b**) flexible strain sensors fabricated by AMJP.

The aforementioned method for printing flexible circuits was used to fabricate strain sensors. As a pre-printing treatment, the PDMS substrate was first treated with plasma cleaning to improve its hydrophilicity and enhance the adhesion during printing. Using silver nanoparticle ink as the printing material, with a sheath gas flow rate of 400 sccm, a carrier gas flow rate of 100 sccm, *L* = 3 mm, *d* = 500 μm, and V = 10 mm/s, the flexible strain sensors were printed on the treated PDMS substrate. After printing, conductive silver paste was applied to both ends of the printed electronic circuit, and copper wires were attached to the silver paste. Finally, the electronic circuit and conductive silver paste were cured through laser micro-sintering. The complete fabrication process is shown in Figure 19a, and the fabricated flexible resistive strain sensors are depicted in Figure 19b.

**Figure 20:**
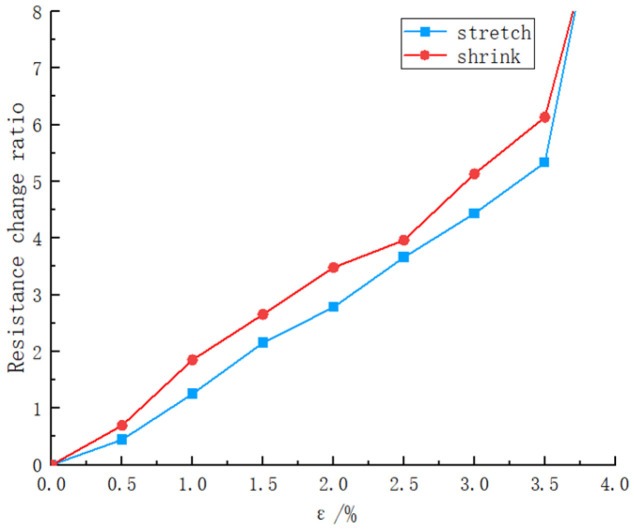
The resistance change rate when stretching and shrinking the strain sensor.

The resistance change of the sensor when subjected to horizontal stretching and contraction strain is shown in Figure 20. The results indicate that the sensor exhibits good linearity within the strain range from 0 to 3.5%, and it is able to reflect the strain state through resistance changes. However, when the strain exceeds 3.5%, the surface micro-cracks of the sensor become too large, resulting in its inability to accurately reflect the measured parameters. During the contraction process of the sensor, due to the time delay in the recovery of micro-cracks, the resistance is larger compared to when it is stretched, exhibiting a certain degree of hysteresis, with a similar pattern of change as during stretching. By considering the working range of the sensor within a strain range from 0 to 3.5% based on the relationship between sensor resistance and strain shown in Figure 19, the sensitivity of the printed sensor with three layers is calculated to be 163.84, indicating good performance. The sensitivity (K) of the resistance-based strain sensor is given by:

(11) $$K=\frac{\Delta R/R}{\varepsilon },$$

where ΔR represents the resistance change of the strain sensor under maximum strain; R represents the base resistance of the sensor; ε represents the maximum strain of the sensor.

**Figure 21:**
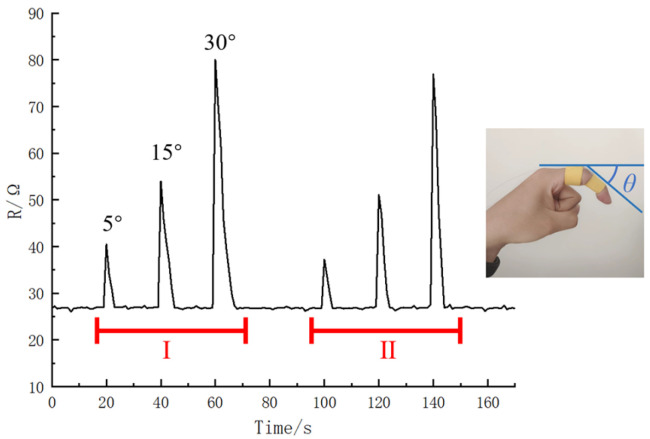
Two test results (I, II) obtained when the fingers are bent at 5°, 15°, and close to 30°.

Performance testing was conducted by fixing the sensor on a finger and using a detector to measure the resistance change when the finger was bent at angles of 5°, 15°, and 30°and then returned to its original position. The tests were carried out twice in a periodic manner, and the results are shown in Figure 21. The test groups I and II represent repeated experiments with three different degrees of bending within a continuous time period. The results reveal that the sensor exhibits similar resistance changes when detecting the same repeated strain, indicating good stability of the flexible strain sensor.

## 4. Discussion

This research delves into the effects of gas flow rate, nozzle diameter, working distance, and printing speed on AMJP, and compares and analyzes the simulation and experimental results. At the same time, uncertain results were also discussed.

In AMJP, the gas flow rate is an important process parameter that directly affects the particle trajectory, which in turn impacts the print quality. The carrier gas flow rate affects the droplet generation and ejection velocity, while the sheath gas flow rate determines the linearity and stability of the aerosol beam. A high carrier gas flow rate can carry more droplets, but it also leads to faster ejection, making it challenging to control the droplet size. Conversely, if the carrier gas flow rate is too low, it may not transport the atomized droplets into the nozzle effectively. An extremely low sheath gas flow rate cannot maintain the shape of the beam, while an extremely high flow rate can cause beam diffusion. Therefore, selecting appropriate gas flow rates can improve the dispersion and transport speed of aerosol particles, thereby enhancing the success rate and accuracy of AMJP.

The nozzle diameter indicates the size of the capillary nozzle at the bottom of the printing nozzle. During the AMJP process, the nozzle diameter can affect the stability of aerosol jetting, printing efficiency, and other characteristics. A smaller nozzle diameter typically enables higher resolution and accuracy, but may limit the flowability of the material, affecting deposition uniformity and continuity, leading to clogging or unstable droplets and impacting the printing quality. On the other hand, a larger nozzle diameter can provide a larger channel for smoother material flow, but may adversely affect the jetting stability and reduce the resolution and accuracy.

The working distance in AMJP refers to the distance between the nozzle exit and the printing substrate. An appropriate working distance is crucial to maintain the straightness and stability of the aerosol jet during the jetting process, enabling high-quality printing. If the working distance is too large, the aerosol jet may disperse or diffuse in the air, affecting the quality of printed lines on the substrate. Conversely, if the working distance is too small, clogging or collisions may occur. Further, when the distance is extremely small, the aerosol jet may splash upon contact with the substrate, resulting in printing defects such as satellite droplets.

The experimental results of this research demonstrate that printing speed has a significant impact on the print quality and efficiency of AMJP. The term “printing speed” mentioned in this research refers to the movement speed of the nozzle during the printing process, which is controlled to regulate the material deposition rate. As the printing speed increases, the material deposition rate also increases, leading to improved production efficiency. However, a higher printing speed tends to result in reduced resolution and increased surface roughness. This can be attributed to the decreased amount of material deposited per unit area, leading to less precise and more uneven printing. Furthermore, it was observed that a slower printing speed allows for better control over the deposition process. This is because a lower speed provides more time for the aerosol particles to settle and adhere to the substrate, resulting in improved adhesion and structural integrity. However, excessively slow movement speeds may lead to prolonged printing times and potential material drying issues.

In addition to the printing parameters discussed in the previous research works, there are several other factors that may influence the print quality and performance. Firstly, the choice of materials used during the printing process can also impact the results. The physical properties of printing materials, including viscosity, surface tension, and rheological properties, may exhibit varying degrees of compatibility with the printing technique, which can affect the formation and deposition of aerosol particles, leading to differences in electrical performance or mechanical stability. Secondly, minor variations in other equipment parameters, such as pressure during aerosolization, nozzle temperature, or laser micro-sintering power, can also have an influence on the printing process. Preliminary explorations were conducted in the initial experiments to address these factors, and some trends and impacts were observed. However, due to limitations in space and experimental conditions, it was not possible to provide a comprehensive discussion and analysis of all potential factors in this study. Additionally, a comparison with previous studies revealed variations in the ranges of printing parameters used, likely due to different printing equipment and material characteristics. Nevertheless, the results of this study consistently demonstrate that appropriate printing parameters are crucial factors for achieving high-quality prints, as supported by previous research [28]. Finally, future investigations will explore alternative explanations or possibilities for any unexpected or uncertain results encountered during the process. By considering alternative hypotheses or scenarios, a more comprehensive analysis and interpretation of the experimental results can be achieved, further refining and optimizing the printing efficiency of AMJP.

## 5. Conclusions

Based on a combination of simulation analysis and experimental verification, the influence of process parameters in AMJP on the minimum feature line width and line morphology of flexible circuits was investigated. The obtained optimal parameters were then applied to print a flexible circuit by stacking 10 layers, enabling the printing of flexible circuits on curved substrates in addition to planar surface printing. This ultimately led to the fabrication of flexible strain sensors. The main findings of the study are summarized as follows:

(1) During the AMJP process, factors such as carrier gas flow rate, sheath gas flow rate, working distance, nozzle diameter, and printing speed had a significant impact on the minimum feature line width of the printed lines. The minimum feature line width gradually decreased with the increase in sheath gas flow rate and printing speed, while it gradually increased with the increase in carrier gas flow rate, working distance, and nozzle diameter.
(2) A discrete phase simulation model was used to predict and optimize the effects of various parameters on the AMJP performance, providing guidance for practical operations. Additionally, through experimental studies, actual printing data were obtained, and the accuracy of the prediction results was validated.
(3) Through single-factor experiments, it was determined that under a carrier gas flow rate of 100 sccm, sheath gas flow rate of 400 sccm, working distance of 3 mm, and nozzle diameter of 500 μm, the minimum feature line width of the printed lines could reach 43 μm, and there were no significant defects such as satellite droplets and deposition voids.
(4) Based on the aforementioned process parameters, flexible strain sensors were fabricated on PDMS substrates, and these sensors were applied to detect mechanical deformations of human fingers. This verified the ability of the sensors to detect subtle changes in human motion, indicating their potential application in fields such as smart wearable devices.

## Figures and Tables

**Table 1:** Single-factor experimental design for AMJP.

X	L (mm)	D (μm)	V (mm/s)
0.5	1	100	6
1	2	300	8
2	3	400	10
3	4	500	12
4	5	600	14
5		800	

## Data Availability

The data presented in this study are available on request from the corresponding author.
